# 2,3-Xylidinium nitrate

**DOI:** 10.1107/S1600536813023465

**Published:** 2013-08-23

**Authors:** Chourouk Kefi, Houda Marouani, Mohamed Rzaigui

**Affiliations:** a Laboratoire de chimie des Matériaux, Faculté des Sciences de Bizerte, 7021 Zarzouna Bizerte, Tunisia

## Abstract

In the crystal structure of the title compound, C_8_H_12_N^+^·NO_3_^−^, the 2,3-xylidinium (2,3-di­methyl­anilinium) cations are connected to the nitrate anions through bifurcated N—H⋯(O,O) and weak C—H⋯O hydrogen bonds, generating corrugated layers parallel to (001) at *z* = 0.25 and 0.75. These layers are connected *via* C—H⋯O inter­actions, giving rise to a three-dimensional network.

## Related literature

For related structures, see: Marouani *et al.* (2010 ▶, 2012 ▶). For graph-set notation of hydrogen-bonding motifs, see: Bernstein *et al.* (1995 ▶).
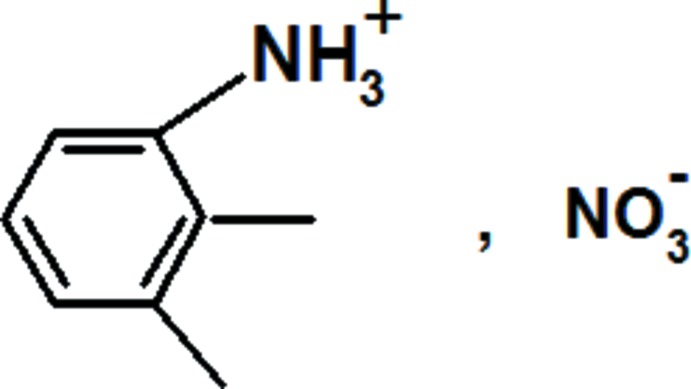


## Experimental

### 

#### Crystal data

C_8_H_12_N^+^·NO_3_^−^
*M* *_r_* = 184.20
Orthorhombic, 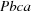

*a* = 10.889 (2) Å
*b* = 10.110 (2) Å
*c* = 17.010 (3) Å
*V* = 1872.5 (6) Å^3^
*Z* = 8
Ag *K*α radiation
λ = 0.56083 Å
μ = 0.06 mm^−1^
*T* = 293 K
0.4 × 0.3 × 0.2 mm

#### Data collection

Enraf–Nonius CAD-4 diffractometer
8719 measured reflections
4541 independent reflections
1933 reflections with *I* > 2σ(*I*)
*R*_int_ = 0.056
2 standard reflections every 120 min intensity decay: 2%

#### Refinement

*R*[*F* ^2^ > 2σ(*F* ^2^)] = 0.060
*wR*(*F* ^2^) = 0.178
*S* = 0.86
4541 reflections
121 parameters
H-atom parameters constrained
Δρ_max_ = 0.13 e Å^−3^
Δρ_min_ = −0.15 e Å^−3^

### 

Data collection: *CAD-4 EXPRESS* (Enraf–Nonius, 1994 ▶); cell refinement: *CAD-4 EXPRESS*; data reduction: *XCAD4* (Harms & Wocadlo, 1995 ▶); program(s) used to solve structure: *SHELXS97* (Sheldrick, 2008 ▶); program(s) used to refine structure: *SHELXL97* (Sheldrick, 2008 ▶); molecular graphics: *ORTEP-3 for Windows* (Farrugia, 2012 ▶) and *DIAMOND* (Brandenburg & Putz 2005 ▶); software used to prepare material for publication: *WinGX* (Farrugia, 2012 ▶).

## Supplementary Material

Crystal structure: contains datablock(s) I. DOI: 10.1107/S1600536813023465/pv2645sup1.cif

Structure factors: contains datablock(s) I. DOI: 10.1107/S1600536813023465/pv2645Isup2.hkl

Supplementary material file. DOI: 10.1107/S1600536813023465/pv2645Isup3.cml

Additional supplementary materials:  crystallographic information; 3D view; checkCIF report

## Figures and Tables

**Table 1:** Hydrogen-bond geometry (Å, °)

*D*—H⋯*A*	*D*—H	H⋯*A*	*D*⋯*A*	*D*—H⋯*A*
N2—H2*A*⋯O3	0.89	2.03	2.915 (2)	177
N2—H2*A*⋯O2	0.89	2.64	3.296 (2)	131
N2—H2*B*⋯O1^i^	0.89	2.11	2.995 (2)	171
N2—H2*B*⋯O2^i^	0.89	2.46	3.148 (2)	134
N2—H2*C*⋯O3^ii^	0.89	2.15	2.973 (2)	153
N2—H2*C*⋯O1^ii^	0.89	2.36	3.158 (2)	149
C4—H4⋯O2^iii^	0.93	2.57	3.439 (3)	156
C7—H7*A*⋯O1^ii^	0.96	2.63	3.522 (3)	155

Symmetry codes: (i) 
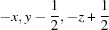
; (ii) 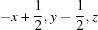; (iii) 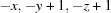.

**Fig. 1.**
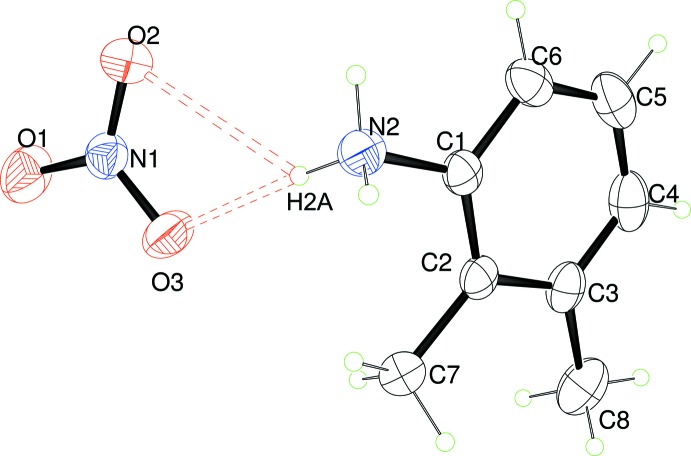
An ORTEP view of (I) with the atom-labelling scheme. Displacement ellipsoids are drawn at the 30% probability level. H atoms are represented as small spheres of arbitrary radii. Hydrogen bonds are shown as dotted lines.

**Fig. 2.**
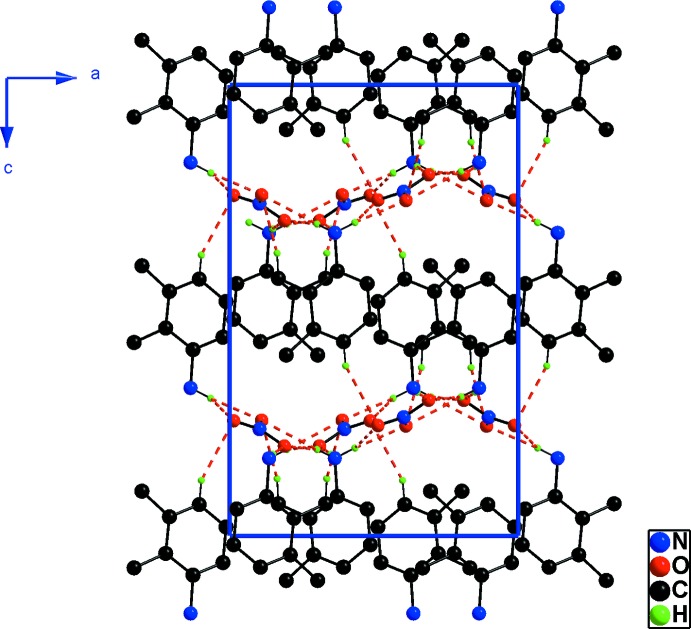
Projection of (I) along the b axis. The H-atoms not involved in H-bonding are omitted.

**Fig. 3.**
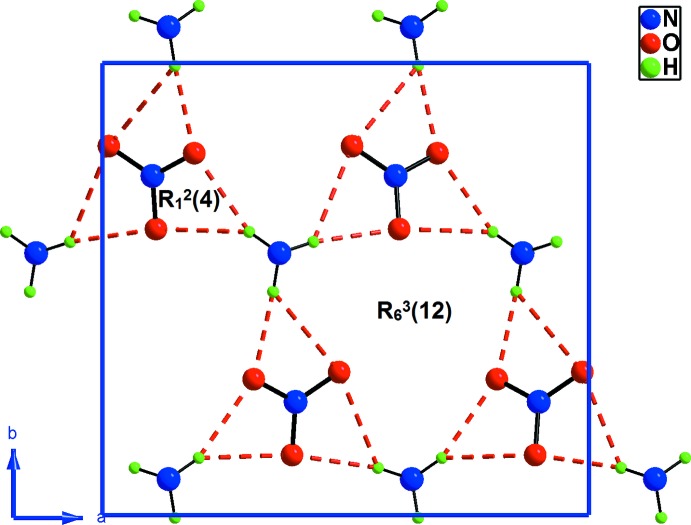
Hydrogen bond motifs in (I).

## supplementary crystallographic information

### 1. Comment

As a part of our study of crystal packing containing the 2,3-xylidinium cation (Marouani, *et al.*, 2010), we report here the preparation and the crystal structure of the title compound (I).

The asymmetric unit of (I) is composed of nitrate anion and 2,3-xylidinium cation (Fig. 1). The bond distances and angles in the anion and the cation agree very well with the corresponding bond distances and angles reported earlier for the anion (Marouani *et al.*, 2012) and the cation (Marouani *et al.*, 2010). The aromatic ring of the cation is essentially planar with an r.m.s deviation of 0.0017 Å. The interplanar distance between the rings of the cations is in the vicinity of 3.569 Å, indicating the formation of π–π interactions.

The cations are connected to the anions through bifurcated N—H···O(O) and weak C7—H7A···O1 hydrogen bonds (Table 1), generating a corrugated layers parallel to the (001) plane at *z* = 0.25 and 0.75 (Fig. 2). These layers are connected *via* C4—H4···O2 interactions, giving rise to a three-dimensional network. Each cation is bonded to three different nitrate anions through six N—H···O hydrogen bonds forming *R*_6_^3^(12) and *R*_1_^2^(4) motifs in the graph-set notation (Fig. 3) (Bernstein *et al.*, 1995). All the hydrogen bonds, the van der Waals contacts, and electrostatic interactions between the different entities give rise to a three-dimensional network in the structure and add stability to the compound.

### 2. Experimental

Single crystals of the title compound were prepared at room temperature from an aqueous mixture of 2,3-xylidine ( 1 mmol) and nitric acid (1 mmol). The mixture was stirred for 15 min then slowly evaporated at room temperature until the formation of good quality pink prismatic single crystals.

### 3. Refinement

All H atoms were fixed geometrically and treated as riding with C—H = 0.93 Å (aromatic) or 0.96 Å (methyl), N—H = 0.89 Å with *U*_iso_(H) = 1.2Ueq(C or N).

### Crystal data

**Table:**

C_8_H_12_N^+^·NO_3_^−^	*F*(000) = 784
*M**_r_* = 184.20	*D*_x_ = 1.307 Mg m^−^^3^
Orthorhombic, *P**b**c**a*	Ag *K*α radiation, λ = 0.56083 Å
Hall symbol: -P 2ac 2ab	Cell parameters from 25 reflections
*a* = 10.889 (2) Å	θ = 8–10°
*b* = 10.110 (2) Å	µ = 0.06 mm^−^^1^
*c* = 17.010 (3) Å	*T* = 293 K
*V* = 1872.5 (6) Å^3^	Prism, pink
*Z* = 8	0.4 × 0.3 × 0.2 mm

### Data collection

**Table:**

Enraf–Nonius CAD-4 diffractometer	*R*_int_ = 0.056
Radiation source: fine-focus sealed tube	θ_max_ = 28.0°, θ_min_ = 2.4°
Graphite monochromator	*h* = −18→3
non–profiled ω scans	*k* = −5→16
8719 measured reflections	*l* = −2→28
4541 independent reflections	2 standard reflections every 120 min
1933 reflections with *I* > 2σ(*I*)	intensity decay: 2%

### Refinement

**Table:**

Refinement on *F*^2^	Primary atom site location: structure-invariant direct methods
Least-squares matrix: full	Secondary atom site location: difference Fourier map
*R*[*F*^2^ > 2σ(*F*^2^)] = 0.060	Hydrogen site location: inferred from neighbouring sites
*wR*(*F*^2^) = 0.178	H-atom parameters constrained
*S* = 0.86	*w* = 1/[σ^2^(*F*_o_^2^) + (0.0586*P*)^2^] where *P* = (*F*_o_^2^ + 2*F*_c_^2^)/3
4541 reflections	(Δ/σ)_max_ < 0.001
121 parameters	Δρ_max_ = 0.13 e Å^−^^3^
0 restraints	Δρ_min_ = −0.15 e Å^−^^3^

### Special details

**Table:**

Geometry. All e.s.d.'s (except the e.s.d. in the dihedral angle between two l.s. planes) are estimated using the full covariance matrix. The cell e.s.d.'s are taken into account individually in the estimation of e.s.d.'s in distances, angles and torsion angles; correlations between e.s.d.'s in cell parameters are only used when they are defined by crystal symmetry. An approximate (isotropic) treatment of cell e.s.d.'s is used for estimating e.s.d.'s involving l.s. planes.
Refinement. Refinement of *F*^2^ against ALL reflections. The weighted *R*-factor *wR* and goodness of fit *S* are based on *F*^2^, conventional *R*-factors *R* are based on *F*, with *F* set to zero for negative *F*^2^. The threshold expression of *F*^2^ > σ(*F*^2^) is used only for calculating *R*-factors(gt) *etc*. and is not relevant to the choice of reflections for refinement. *R*-factors based on *F*^2^ are statistically about twice as large as those based on *F*, and *R*- factors based on ALL data will be even larger.

### Fractional atomic coordinates and isotropic or equivalent isotropic displacement parameters (Å^2^)

**Table:**

	*x*	*y*	*z*	*U*_iso_*/*U*_eq_	
N1	0.10288 (17)	0.74955 (19)	0.26311 (10)	0.0625 (5)	
O2	0.01325 (15)	0.68356 (15)	0.24486 (9)	0.0858 (6)	
O3	0.18890 (15)	0.69932 (14)	0.30086 (10)	0.0812 (5)	
O1	0.11018 (16)	0.86708 (16)	0.24375 (10)	0.0992 (6)	
C2	0.22220 (17)	0.44029 (17)	0.46097 (12)	0.0523 (5)	
C1	0.12753 (19)	0.39756 (18)	0.41282 (12)	0.0517 (5)	
N2	0.13609 (14)	0.42040 (15)	0.32795 (10)	0.0613 (5)	
H2A	0.1490	0.5060	0.3189	0.092*	
H2B	0.0663	0.3956	0.3050	0.092*	
H2C	0.1981	0.3735	0.3084	0.092*	
C3	0.2094 (2)	0.4186 (2)	0.54217 (13)	0.0629 (6)	
C6	0.0239 (2)	0.33705 (19)	0.44060 (15)	0.0668 (6)	
H6	−0.0381	0.3111	0.4063	0.080*	
C7	0.33189 (17)	0.5102 (2)	0.42887 (14)	0.0669 (6)	
H7A	0.3361	0.4968	0.3731	0.100*	
H7B	0.4048	0.4756	0.4531	0.100*	
H7C	0.3254	0.6030	0.4398	0.100*	
C4	0.1061 (2)	0.3555 (2)	0.56938 (15)	0.0788 (7)	
H4	0.0986	0.3394	0.6230	0.095*	
C5	0.0131 (2)	0.3153 (2)	0.51969 (16)	0.0792 (8)	
H5	−0.0564	0.2738	0.5398	0.095*	
C8	0.3045 (2)	0.4673 (3)	0.59902 (15)	0.0933 (8)	
H8A	0.3094	0.5619	0.5962	0.140*	
H8B	0.3828	0.4297	0.5859	0.140*	
H8C	0.2821	0.4413	0.6514	0.140*	

### Atomic displacement parameters (Å^2^)

**Table:**

	*U*^11^	*U*^22^	*U*^33^	*U*^12^	*U*^13^	*U*^23^
N1	0.0656 (11)	0.0694 (12)	0.0525 (10)	0.0014 (12)	0.0011 (10)	0.0037 (10)
O2	0.0669 (10)	0.1033 (14)	0.0872 (12)	−0.0192 (10)	−0.0084 (9)	0.0135 (9)
O3	0.0770 (10)	0.0763 (11)	0.0902 (12)	0.0059 (9)	−0.0291 (10)	0.0099 (9)
O1	0.1262 (15)	0.0573 (9)	0.1142 (14)	−0.0031 (11)	−0.0345 (13)	0.0181 (10)
C2	0.0550 (13)	0.0402 (11)	0.0615 (13)	0.0111 (10)	−0.0002 (10)	0.0052 (10)
C1	0.0564 (12)	0.0385 (10)	0.0601 (13)	0.0065 (10)	0.0027 (11)	0.0020 (9)
N2	0.0620 (11)	0.0539 (10)	0.0681 (12)	0.0002 (9)	−0.0070 (9)	−0.0017 (9)
C3	0.0766 (16)	0.0531 (13)	0.0590 (14)	0.0207 (12)	0.0058 (12)	0.0057 (11)
C6	0.0624 (14)	0.0465 (12)	0.0915 (17)	−0.0005 (12)	0.0080 (13)	−0.0024 (12)
C7	0.0590 (13)	0.0721 (14)	0.0696 (14)	−0.0010 (12)	−0.0089 (11)	0.0076 (12)
C4	0.104 (2)	0.0593 (15)	0.0731 (16)	0.0218 (15)	0.0272 (17)	0.0126 (13)
C5	0.0821 (18)	0.0518 (15)	0.104 (2)	0.0026 (13)	0.0325 (16)	0.0089 (14)
C8	0.111 (2)	0.103 (2)	0.0656 (15)	0.0249 (18)	−0.0158 (15)	−0.0014 (14)

### Geometric parameters (Å, º)

**Table:**

N1—O2	1.222 (2)	C3—C8	1.500 (3)
N1—O1	1.236 (2)	C6—C5	1.368 (3)
N1—O3	1.244 (2)	C6—H6	0.9300
C2—C1	1.386 (3)	C7—H7A	0.9600
C2—C3	1.406 (3)	C7—H7B	0.9600
C2—C7	1.491 (3)	C7—H7C	0.9600
C1—C6	1.368 (3)	C4—C5	1.381 (3)
C1—N2	1.465 (2)	C4—H4	0.9300
N2—H2A	0.8900	C5—H5	0.9300
N2—H2B	0.8900	C8—H8A	0.9600
N2—H2C	0.8900	C8—H8B	0.9600
C3—C4	1.373 (3)	C8—H8C	0.9600
			
O2—N1—O1	120.6 (2)	C5—C6—H6	120.6
O2—N1—O3	120.63 (19)	C2—C7—H7A	109.5
O1—N1—O3	118.80 (19)	C2—C7—H7B	109.5
C1—C2—C3	117.3 (2)	H7A—C7—H7B	109.5
C1—C2—C7	121.81 (19)	C2—C7—H7C	109.5
C3—C2—C7	120.9 (2)	H7A—C7—H7C	109.5
C6—C1—C2	123.3 (2)	H7B—C7—H7C	109.5
C6—C1—N2	117.6 (2)	C3—C4—C5	122.1 (2)
C2—C1—N2	119.08 (18)	C3—C4—H4	118.9
C1—N2—H2A	109.5	C5—C4—H4	118.9
C1—N2—H2B	109.5	C6—C5—C4	119.4 (2)
H2A—N2—H2B	109.5	C6—C5—H5	120.3
C1—N2—H2C	109.5	C4—C5—H5	120.3
H2A—N2—H2C	109.5	C3—C8—H8A	109.5
H2B—N2—H2C	109.5	C3—C8—H8B	109.5
C4—C3—C2	119.0 (2)	H8A—C8—H8B	109.5
C4—C3—C8	120.0 (2)	C3—C8—H8C	109.5
C2—C3—C8	120.9 (2)	H8A—C8—H8C	109.5
C1—C6—C5	118.9 (2)	H8B—C8—H8C	109.5
C1—C6—H6	120.6		

### Hydrogen-bond geometry (Å, º)

**Table:**

*D*—H···*A*	*D*—H	H···*A*	*D*···*A*	*D*—H···*A*
N2—H2*A*···O3	0.89	2.03	2.915 (2)	177
N2—H2*A*···O2	0.89	2.64	3.296 (2)	131
N2—H2*B*···O1^i^	0.89	2.11	2.995 (2)	171
N2—H2*B*···O2^i^	0.89	2.46	3.148 (2)	134
N2—H2*C*···O3^ii^	0.89	2.15	2.973 (2)	153
N2—H2*C*···O1^ii^	0.89	2.36	3.158 (2)	149
C4—H4···O2^iii^	0.93	2.57	3.439 (3)	156
C7—H7*A*···O1^ii^	0.96	2.63	3.522 (3)	155

Symmetry codes: (i) −*x*, *y*−1/2, −*z*+1/2; (ii) −*x*+1/2, *y*−1/2, *z*; (iii) −*x*, −*y*+1, −*z*+1.
